# A comparison of general health and coping strategies in fertile and infertile women in Yazd 

**Published:** 2012-11

**Authors:** Ali Reza Bakhshayesh, Mahsa Kazeraninejad, Mahsa Dehghan Mongabadi, Malihe Raghebian

**Affiliations:** *Department of Psychology and Education, Faculty of Humanities, Yazd University, Yazd, Iran.*

**Keywords:** *General health*, *Coping strategies*, *Fertility and infertility*

## Abstract

**Background: **Infertility affects various aspects of personality and psychology, familial and career performances, and relationships. Studies show that stress, anxiety, life dissatisfaction, and other psychological problems follow infertility. Infertility issue, its tests and remedy are stressful and may lead to anxiety and depression and have destructive effects on couple relationships.

**Objective:** The present study was done in order to comparison general health and coping strategies in fertile and infertile women.

**Materials and Methods:** This is an analytic cross-sectional study and was done through random sampling on 70 fertile women and 70 infertile women who visited Yazd’s clinics. The age range of participants was between 20-40 years. General Health Questionnaire (GHQ) and Ways of coping questionnaire (WOCQ) were filled by women who were agreed to participate in the study, following some explanations about aims and ways of doing the study. In the next step, data were analyzed through statistic methods and independent t-test. We considered a significant level p<0.05 in all tests.

**Results:** The results indicated a significant difference (p<0.05) with respect to general health in two groups, but no significant difference was found in problem-centered and emotion centered coping strategies and depression anxiety.

**Conclusion:** This study shows that general health in both groups is below average which means women are not sensitive about their general health. So planning on improving women’s general health by providing consultation and training courses is suggested.

## Introduction

In today’s modern world, the problem of infertility is increasing and becoming a social concern. Infertility was described as a global sanitation problem with physical, psychological, and emotional aspects in Bangkok International Conference in 1988 (1). Infertility and its remedies are major crises in one’s life that can prolong several years. “This incident provokes tension in life and couples are subjected to an emotional attack” (2). As Stage mentioned (2007), the uncertainty associated with infertility is the key challenge for infertile women (3).

With respect to these processes, it can be implied that any good or bad changes in life requires a type of further compatibility. Strategies to encounter these changes in life and the resultant tension vary in different individuals regarding different situations (4). Author’s views in coping definition are arranged on a continuum with two opposite poles in two sides; in one hand it is believed that coping means how people overcome their problems (5) and in two other poles there is Folkman and Lazarus theory which defines coping stressful, regarding environmental conditions and environment management quality (6). “Coping strategies are collection of one’s cognitive and behavioral efforts which are used to interpret, analyze, and reform a stressful condition, resulting in the reduction of its discomfort” (4).

Two main coping strategies are: emotional coping strategies, including efforts to set emotional consequences of stressful incident and keep the emotional and sentimental balance by controlling resultant emotions from stressful conditions; and problem centered coping strategies including one’s effective acts with respect to stressful conditions, and is trying to remove or change the source of stress (4). Dynamism is known as the common feature of problem-centered coping strategies. Dynamism provides requisite equipment's for active coping with stressful situations. This condition needs person’s all potential abilities for opposite coping and solving the problem, and increases his success probability (7). 

Another feature of people who use problem-centered coping strategies is the low level of tension. On the other side, denial and passivity are two characteristics of those who ineffective emotion-centered coping strategies. Denial of stressful situations and inability to use potential abilities and initiative will cause the occurred problem left unsolved, which leads to dissatisfaction of the person (7). 

Moller *et al* have found that psychic pressures and worries resulting from infertility have a direct effect on physiological functions of body which ultimately leads to a negative effect on fertility (8). Omoaregba *et al* (2011), indicated that "the prevalence of probable psychological distress was significantly higher among the infertile group compared with their fertile counterparts (p<0.001)" (9). 

Daniel *et al* (2010), have the same idea, and mention that, compared with fertile women, infertile women have higher odds of self-reported depression (10). Substantial evidences point that problems resulting from infertility and inappropriate coping strategies might be a factor which helps exacerbate infertility (11). However, Bringer *et al* (2010) provide evidence against the hypothesis, casual relationship between mental distress and infertility (12).

On the other side, those people who have peace of mind and general health, experience less psychic pressure, consequently their chance of fertility increase (11). Depression is one of the main psychological problems in infertile individuals, which has a noticeable impression on all aspects of their life (12). As Peterson *et al* (2010) have reported in their study, infertility stress which is linked with depression and psychological distress, can lead to premature dropout from medical treatments and unresolved feelings of loss and grief (13).

Hariiyan, Mohammadpur, and Aghajanlu have found that 58% of 100 infertile women, to some extent, are suffering from depression (12). Litt *et al* reported that "25% of infertile women will get depression following IVF unsuccessful treatment" (14, 15). Infertile couples experience the resultant tension of depression individually and cooperatively. Such tension may lead to mental problems (16, 17).

Ramezanzadeh (2004) has shown that, in comparison to fertile women, infertile women experience more anxiety and depression (18). Aghanwa, Dare, and Oguniy in 1999, reported in their research that "7.29% of infertile women suffer from depression and anxiety disorder" (19). In fact, psychological factors not only can cause infertility, but also can lead to different psychological issues (18).

With respect to the fact that, not only Yazd province is one of the poles of infertility in Iran, and in Iranian culture, ability to become fertile has a great significant, but also, so far, no research was done with the subject of both coping strategies, and general health of fertile and infertile women.

Therefore this research was done to examine general health and coping strategies in fertile and infertile women who visited Yazd clinics, and tried to answer the following questions: 

1. Is there a difference in emotional-centered coping strategies scale in fertile and infertile women? 

2. Is there a difference in problem-centered coping strategies scale in fertile and infertile women? 

3. Is there a difference in general health in fertile and infertile women? 

## Materials and methods

The strategy for this study was analytic cross-sectional and required information was collected from fertile and infertile women by Ways of Coping Questionnaire (WOCQ) and General Health Questionnaire (GHQ). Statistical society in the present research was all fertile and infertile women conferring to women’s clinics of Yazd.

In order to choose sample, fertile and infertile women (in the age range of 20 to 40 years) who conferred to women’s clinics were chosen by simple random sampling. There were 140 participants including 70 fertile and 70 infertile women, and 133 questionnaires were returned. Results were analyzed by SPSS statistics software and independent t-test.

**Measurement tools**

General Health Questionnaire (GHQ): This test is designed by Goldberg and consists of 28 questions that evaluate 4 factors: physical, anxiety, social function disorder and depression. So far, more than 70 researches have been done on validity of GHQ all over the world. Goldberg and Marry in 1987 (20) have reported that average sensibility of the questionnaire is 82% and the result of validity has shown that sensibility and feature of this questionnaire in the best cut-off score are 86.5% and 82% respectively, and the final coefficient with retest and Cronbach’s alpha is 88%, which is in 99% trust level (21).

Ways of Coping Questionnaire (WOCQ): this is a 66 article test which is built with respect to Lazarus and Folkman’s inventory, by themselves, and evaluates a wide range of thoughts and deeds that are done when the person is confronted to external and internal stressful situations. At first, participants were asked to describe recently experienced stressful situation, oral or written, and then, by reading the questionnaire, specify how he/she has used each of the following strategies; this test contains 7 subscales: 1) direct coping, 2) going away, 3) self-controlling, 4) social sensitivity demand, 5) responsibility, 6) escape-avoidance, and 7) solving the problem.

**A. Problem centered strategies**

Social support demand: describes efforts seeking informational and emotional support (questions: 8, 18, 22, 31, 42, 45). 

Responsibility: accepting self-role in problem which is always accompanied by an effort to place everything in the right place (questions: 9, 25, 29, 51). 

Thoughtfully solving the problem describes thoughtful problem-centered efforts for changing the situation which is followed by solving the problem (questions: 1, 26, 39, 48, 49, 52).

Double positive evaluation: efforts which adds positive meaning by focusing on personal growth. This scale has religious concept. (questions: 20, 23, 30, 36, 38, 56, 60)

**B. Emotion-centered strategies**

Encounter coping: describes aggressive efforts done to change the situation and has some levels of hostility and risk-taking (questions: 6, 7, 17, 28, 34, 46).

Avoidance: cognitive efforts to make yourself alone and minimizing the importance of situation (questions: 44, 41, 21, 15, 13, 12(.

Continence: describes efforts which set one's emotions and actions. (questions: 10, 14, 35, 43, 54, 62, 63).

Escape: describes dream thoughts and cognitive efforts in order to avoid the problem. This scale is different from avoidance scale (questions: 11, 16, 33, 40, 47, 50, 58, 59). Lazarus and Folkman (1988) reported internal stability =0.66-0.79 through Cronbach's Alpha for all coping strategies.

**Statistical analysis**

In order to analyze information in this research, descriptive statistic (mean, standard deviation) and independent t-test were used.

## Results

**Figure 1:**
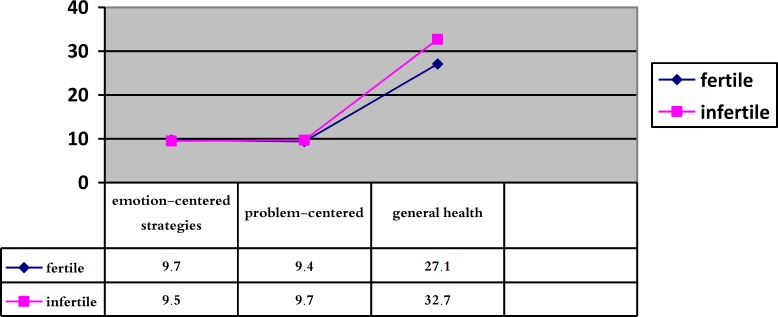
A comparison between mean in fertile and infertile groups

This research evaluates emotion-centered strategies, problem-centered strategies and psychological health in fertile and infertile groups. The values of all variables are listed below (Table I, Figure 1). 

Our findings showed that the two groups have no significant differences in using coping strategies. The mean of the resultant data in emotion-centered coping strategies in fertile women was 9.7±3.5, and in infertile women was 9.5±1.14. The mean of the resultant data in problem-centered coping strategies in fertile women was 9.4±2.19 and in infertile women was 9.7±1.65. The results of independent t-test showed no significant differences between two groups in coping strategies (p=0.6). However, we can notice such difference in the case of their general health (p=0.004). 

**Table I:** Mean, standard deviation, maximum and minimum score in coping strategies and psychological health and its derivative subtests in fertile and infertile women

**Variable**	**Emotion-centered strategies**	**Problem-centered strategies**	**Psychological health**
Number of tests	133	133	133
Mean	9.6	9.6	29.9
Standard deviation	2.6	1.9	11.5
Maximum score	26	20.1	57
Minimum score	*6.5*	*5.7*	4

## Discussion

Is there a difference in emotional-centered coping strategies scale in fertile and infertile women? This was the first question of this study. Results show that there is no difference in emotion-centered coping strategies in fertile and infertile women, which means that the level of using emotion-centered coping strategies in fertile and infertile women is almost the same. In this aspect, this study is the same as Pahlavani *et al *research. In their study, 40 fertile (20 males, 20 females) and 40 infertile (20 males, 20 females) were compared. 

They used SCL-90-R and Carver-Schier coping skills check list. Pahlavani *et al* (2002) got to this point that there is no difference in using emotion-centered coping strategies in fertile and infertile women (22). But the result is not the same in Shakeri’s research (2006). In their study 150 infertile women conferring to infertility clinics were studied and data were collected through GHQ-28 general health questionnaire and CRI coping responses. It shows that most of the infertile women use emotion-centered strategy (11). 

Panagopoulou *et al* (2009) "do not support the hypotheses that emotional disclosure will reduce infertility-related psychological distress". In their study, "148 participants were randomized to an emotional-writing condition, a fact-writing condition and a control condition"(23).

The second question was: Is there a difference in problem-centered coping strategies scale in fertile and infertile women? Results show that there is no difference in using problem-centered coping strategies in fertile and infertile women. Despite the fact that we tried to find a study in this field, no study was found to compare the results.

Next question was: Is there a difference in general health in fertile and infertile women? Results show that there is a significant difference in fertile and infertile women with respect to general health. 

General health in fertile women was in higher level than infertile women. In this aspect, our results were the same as Besharat *et al* in 2006 (24). In their research 45 fertile and 45 infertile women conferring to infertility clinics were studied and data were collected through the Golombok Rust Inventory of Marital State and Veit-Ware Mental health Inventory. They have got to the point that general health in fertile women stands in a higher level than infertile women. 

Similarly, the same results were obtained by Bahrami *et al* (2007). In their study 150 fertile and 150 infertile couples were studied and Beck depression inventory and Larson sexual satisfaction inventory were used.

## Conclusion

This study shows that tension and worry effect general health in infertile women and reduce it. But there are no significant difference in anxiety and depression and using emotion centered and problem-centered coping strategies in fertile and infertile women.
